# Associations between Life’s Essential 8 and gallstones among US adults: A cross-sectional study from NHANES 2017–2018

**DOI:** 10.1371/journal.pone.0312857

**Published:** 2024-10-30

**Authors:** Ting Wang, Ruijie Xie, Cong Jiang, Lanyu Chen

**Affiliations:** 1 Department of Infectious Diseases, Guang ’anmen Hospital of China Academy of Chinese Medical Sciences, Beijing, China; 2 Department of Hand & Microsurgery, The Affiliated Nanhua Hospital, Hengyang Medical School, University of South China, Hengyang, China; 3 Department of Diabetic Nephrology, Muping District Traditional Chinese Medicine Hospital, Shandong, China; King’s College Hospital NHS Trust: King’s College Hospital NHS Foundation Trust, UNITED KINGDOM OF GREAT BRITAIN AND NORTHERN IRELAND

## Abstract

**Background:**

Cardiovascular illness and gallstones are closely related. Our goal was to determine whether gallstones and the updated LE8 score, which measures cardiovascular health among US adults, are related.

**Methods:**

3,570 adults participated in the 2017–2018 National Health and Nutrition Examination Survey, which provided the data for our study. Based on the criterion provided by the American Association for Cardiovascular Health (AHA), LE8 score (range 0–100) was calculated and classified as low (0–49), moderate (50–79), and high (80–100) cardiovascular health. Gallstones were derived from the questionnaire. Multivariate logistic modeling explored the independent relationship between LE8 score and gallstones.

**Results:**

There was a negative correlation between LE8 score and gallstones. Specifically, the odds of gallstones dropped by 15% for each 10-unit increase in LE8 score (OR = 0.85; 95% CI, 0.77–0.94). Smooth curve fitting detected a saturation effect between LE8 score and gallstones, with a minimum threshold of 66.25 points associated with both. There was a noticeably stronger inverse relationship between gallstones and LE8 score in those under 60 years of age and not taking antihypertensive or lipid-lowering drugs.

**Conclusions:**

Lower LE8 scores may be a potential risk factor for the development of gallstones and could also be a target for risk assessment and intervention.

## Introduction

Gallbladder and bile duct diseases (GBDs) are among the most common digestive disorders [1]. Gallstones are among the most prevalent and a common cause of complex biliary disorders [2, 3], impacting around 10–20% of adults globally [4], and are the most common digestive disorder requiring hospitalization in the West [5]. Gender, age, genetics, obesity, and insulin resistance are widely recognized risk factors for gallstones [6–8]; the odds of gallstones have been steadily increasing in recent years with the increase in unhealthy lifestyles such as excess nutritional intake, overnight stays and lack of exercise. Despite considerable current research on the pathogenesis and genetics of gallstones, treatment options are still dominated by invasive treatments, which impose a financial burden on patients and cause persistent gastrointestinal symptoms in 40% of them, making it a mediocre choice of treatment for patients with simple gallstones. Therefore, proactively exploring preventive strategies is of great importance in reducing the public health and economic burden of gallstones.

Assessment of cardiovascular health status is an effective means of monitoring the health of individuals and populations while demonstrating great potential as a primordial prevention strategy to improve and prolong lives [9]. As a result, the American Heart Association (AHA) initially introduced the "Life’s Simple 7" (LS7) score in 2010 as a thorough and quantitative method to evaluate individual’s and populations’ cardiovascular health [10]. However, as the study progressed, the differences and sensitivity of CVH defined by LS7 to individuals were lower than expected. To revise and refine the algorithm for the CVH assessment, AHA incorporated the " Life’s Essential 8" (LE8) score in 2022 [11]. LE8 score employs a continuous scale (range 0 to 100), updates the definition of some sub-scores based on LS7, and introduces sleep health as the evaluation criterion for the first time, which captures individual health behaviors and practices more comprehensively [12]. The association between CVH assessed by the newly defined LE8 score and different clinical outcomes has been extensively studied, with higher CVH showing an independent negative correlation with diseases such as NAFLD, chronic kidney disease, and depression, in addition to lower cardiovascular risk [13–15].

It is well known that metabolic diseases are defined as a collection of multiple cardiovascular risk factors [16]. According to the survey, patients with gallstones have a higher cumulative mortality rate due to cardiovascular disease; in addition, the presence of gallstones is also linked to an elevated odds of cardiovascular disease [17]. Consequently, early cardiovascular health intervention may contribute to the prevention and protection against gallstones. Although previous studies have demonstrated an association between gallstones and individual metabolism-related risk factors (such as physical activity and diet) [18, 19], the possible impact of CVH metrics on gallstones remains unclear. Therefore, the purpose of our study was to look into the relationship between gallstones and CVH levels (as determined by LE8 score) in adult individuals from the National Health and Nutrition Examination Survey database in the United States.

## Materials and methods

### Study population

The National Center for Health Statistics (NCHS) conducted a nationwide cross-sectional survey called NHANES, from which the research’s data were taken and designed to provide statistical data on health-related issues through laboratory testing, physical examinations, and interviews with people in the United States. NHANES utilizes a sophisticated multi-stage probability sampling methodology for the entire U.S. population to represent the samples well. All participants provided written informed consent. The Centers for Disease Control and Prevention and the National Center for Health Statistics’ Research Ethics Review Board gave their approval to the survey protocol.

**Fig 1:**
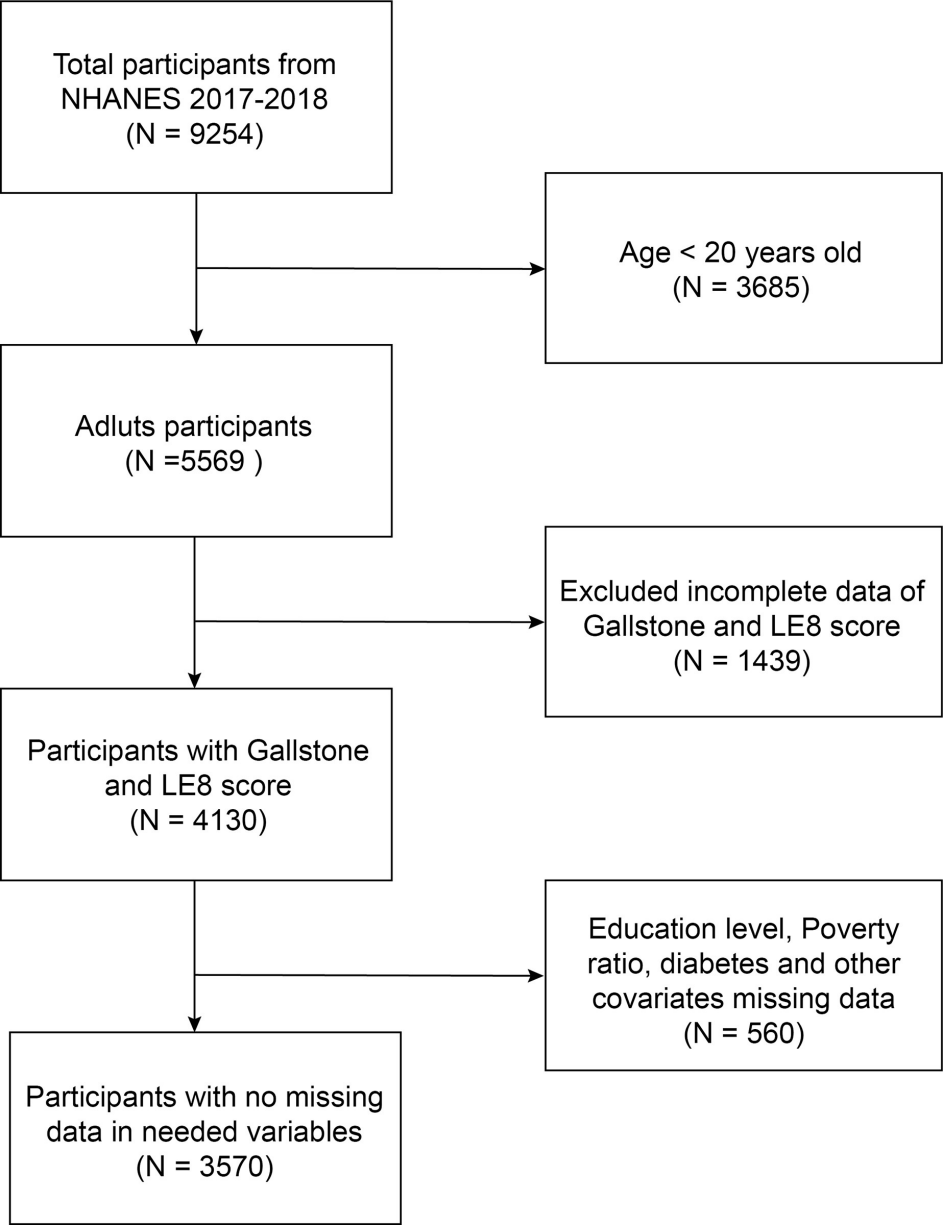
Flow chart showing the quantity of NHANES participants included in the current research.

We collected data on 9,254 subjects from the NHANES database during 2017–2018, and after excluding subjects aged <20 years (n = 3685), missing data on gallstones and LE8 score (n = 1439), and missing data on relevant covariates (n = 560), 3,570 participants were ultimately enrolled in the current study (Fig 1).

### Exposure and outcome variable definitions

LE8 score was designed as the exposure variable, which was calculated from eight sub-scores comprising four health behaviors (DASH diet score, physical activity score, tobacco exposure score, and sleep health score) and four health factors (body mass index score, blood lipid score, blood glucose score, and blood pressure score). Every sub-score ranges from 0–100, and LE8 score, an unweighted average of the 8 sub-scores, also ranges from 0–100, with higher values indicating better cardiovascular health. Participants’ CVH was categorized as low (LE8 score < 50), medium (50 ≤ LE8 score < 79), and high (LE8 score > 79) according to the recommendations of the American Cardiovascular Society [11]. Detailed instructions for calculating LE8 score are available in S1 Table.

The outcome variable was gallstones, and gallstone status was obtained from the 2017–2018 NHANES Health Questionnaire, derived from whether a physician or other health professional had ever informed the subject of having gallstones.

### Covariables

Potential covariates in multivariate correction models may confound the association between LE8 score and gallstones. This study included gender, age, race, education level, marital status, poverty ratio, diabetes (yes/no), cancer (yes/no), cardiovascular disease (yes/no), and use of lipid-lowering or antihypertensive medications (yes/no) as covariates. The study variables’ comprehensive measurement protocols are accessible to the general public at www.cdc.gov/nchs/nhanes/.

### Statistical analysis

Eligible NHANES sample weights were used in all statistical analyses, which were executed in compliance with CDC recommendations. Continuous variables were expressed as weighted means, and categorical variables were expressed as weighted percentages, with corresponding confidence intervals (CIs). For categorical data, the Rao-Scott chi-square test was used to assess baseline characteristics, and for continuous variables, unadjusted linear regressions were used. Weighted multivariate logistic regression analysis examined the association between LE8 score and gallstones in three models. Model 1 did not adjust for covariates; Model 2 adjusted for gender, age, race, education level, marital status, and poverty ratio; Model 3 adjusted for all covariates. A weighted generalized summation model and smoothed curve fitting were used to further assess whether there was a nonlinear association between LE8 score and gallstones, and threshold effect analysis was used to find inflection points. Finally, subgroup analyses stratified by sex, age, diabetes, cardiovascular disease, cancer, and whether or not they were taking lipid-lowering or antihypertensive medications were also performed using multifactorial regression analysis. Meanwhile, an additional subgroup analysis was conducted in the female population adjusted for the parity status. Additionally, a log-likelihood ratio test model was used to test for heterogeneity of connections between subgroups by adding an interaction factor. All data analyses were performed using EmpowerStats version 4.1 and R version 4.0.3 software; p < 0.05 was considered statistically significant.

## Results

### Baseline characteristics

3570 participants aged 20 years and older with complete data were incorporated into this research. Weighted demographic baseline characteristics stratified by the NAFLD status classification were condensed in Table 1. The weighted mean age among the participants was 48.8 years (95%CI, 47.5–50.1), and the proportion of females was 51.3% (95%CI, 48.7–54.0). The mean LE8 score was 68.1 (95%CI, 66.9–69.4), and the percentages of low, moderate, and high CVH defined by LE8 score were 10.1% (95%CI, 9.0–11.3), 66.3% (95%CI, 63.2–69.2) and 23.6% (95%CI, 20.2–27.5) respectively. Besides, 411 individuals received a gallstone diagnosis. Compared with those without gallstones, participants with gallstones were older, more likely to be female, more likely to be separated, and more likely to be taking lipid-lowering or blood pressure medications. They had a higher body mass index, less activity, and poor blood sugar and blood pressure control. No significant differences were observed in race, education, poverty, DASH diet score, sleep health, and lipid status. Rates of DM, cancer, and CVD were about twice as high in those with gallstones, and those without gallstones had higher LE8 scores.

**Table 1:** Basic characteristics of participants (n = 3570) in the NHANES 2017–2018.

	Overall	Non-GALLSTONE	GALLSTONE	*P*-value
	N = 3570	N = 3159	N = 411	
Age(years)	48.8 (47.5, 50.1)	47.7 (46.3, 49.1)	56.7 (55.3, 58.1)	<0.001
Age strata				<0.001
20–59	69.9 (65.5, 73.9)	72.5 (67.4, 77.6)	51.0 (45.0, 56.8)	
≥ 60	30.1 (26.1, 34.5)	27.5 (23.5, 31.8)	49.1 (43.2, 55.0)	
Gender(%)				<0.001
Male	48.7 (46.0, 51.3)	51.4 (48.8, 54.1)	28.8 (23.8, 34.4)	
Female	51.3 (48.7, 54.0)	47.9 (46.0, 51.2)	71.2 (65.6, 76.2)	
Race(%)				0.112
Mexican American	7.8 (5.2, 11.6)	8.0 (5.3, 12.0)	6.1 (3.9, 9.5)	
Other Hispanic	6.5 (5.1, 8.2)	6.7 (5.1, 8.7)	5.3 (2.7, 10.1)	
Non-Hispanic White	65.7 (59.8, 71.1)	64.8 (58.4, 70.6)	72.2 (63.4, 79.5)	
Non-Hispanic Black	10.6 (7.7, 14.5)	11.2 (8.0, 15.5)	6.2 (4.3, 9.1)	
Other Races	9.4 (7.3, 12.0)	9.3 (7.1, 12.0)	10.2 (5.7, 17.6)	
Poverty ratio				0.060
< 1.3	16.7 (15.3, 18.3)	16.7 (15.0, 18.4)	17.1 (12.2, 23.4)	
1.3–3.5	32.9 (29.0, 37.1)	31.8 (27.6, 36.2)	41.4 (32.2, 51.2)	
> 3.5	41.9 (37.3, 46.6)	43.1 (38.3, 48.1)	33.1 (26.8, 40.1)	
Unclear	8.4 (7.0, 10.1)	8.4 (6.7, 10.5)	8.4 (5.0, 13.7)	
Marital status				0.006
Coupled	63.4 (60.1, 66.5)	63.4 (60.2, 66.5)	62.9 (55.1, 70.1)	
Widowed or separated	18.5 (16.4, 20.7)	17.6 (15.5, 19.9)	25.0 (19.0, 32.1)	
Never married	18.2 (15.7, 20.9)	19.0 (16.3, 22.0)	12.1 (8.1, 17.6)	
Education level (%)				0.770
Less than high school	9.3 (7.8, 11.0)	9.3 (7.7, 11.2)	9.0 (6.4, 12.4)	
High school or GED	27.1 (23.8, 30.6)	26.8 (23.1, 30.8)	29.1 (22.2, 37.2)	
Above high school	63.7 (59.2, 67.9)	63.9 (59.0, 68.6)	61.9 (54.6, 68.6)	
Diabetes (%)				<0.001
Yes	12.0 (10.7, 13.4)	10.6 (9.2, 12.0)	22.7 (17.0, 29.7)	
No	88.0 (86.6, 89.3)	89.5 (88.0, 90.8)	77.3 (70.3, 83.0)	
Cancer (%)				0.027
Yes	11.2 (9.8, 12.8)	10.4 (8.8, 12.2)	17.1 (11.3, 25.1)	
No	88.8 (87.2, 90.2)	89.6 (87.8, 91.2)	82.9 (74.9, 88.7)	
Cardiovascular disease (%)				0.016
Yes	4.6 (3.2, 6.6)	4.2 (2.9, 6.1)	7.5 (4.2, 13.0)	
No	95.4 (93.4, 96.8)	95.8 (93.9, 97.1)	92.5 (87.0, 95.8)	
Taking anti-hypertensive or lipid-lowering medicine (%)				<0.001
Yes	34.3 (32.1, 36.6)	31.9 (30.0, 33.9)	51.5 (45.1, 57.8)	
No	65.7 (63.4, 67.9)	68.1 (66.1, 70.0)	48.5 (42.2, 54.9)	
LE8 scores (out of 100 possible points				
LE8 score	68.1 (66.9, 69.4)	68.9 (67.7, 70.1)	62.9 (60.9, 64.9)	<0.001
DASH diet score	38.1 (35.2, 41.0)	38.1 (35.1, 41.1)	38.1 (33.8, 42.4)	0.989
Physical activity score	76.4 (74.3, 78.4)	77.4 (75.3, 79.6)	68.5 (63.9, 73.1)	0.003
Tobacco exposure score	73.8 (71.4, 76.3)	74.0 (71.4, 76.7)	72.2 (67.4, 76.9)	0.483
Sleep health score	83.7 (82.4, 85.1)	83.9 (82.4, 85.5)	82.2 (79.1, 85.3)	0.364
Body mass index score	56.1 (53.3, 58.9)	57.8 (55.0, 60.5)	43.9 (39.2, 48.6)	<0.001
Blood lipids score	67.1 (64.6, 69.7)	67.5 (64.8, 70.2)	64.6 (61.3, 68.0)	0.117
Blood glucose score	83.3 (82.2, 84.3)	84.4 (83.3, 85.4)	75.2 (71.6, 78.7)	<0.001
Blood pressure score	66.7 (64.7, 68.7)	67.8 (65.7, 69.9)	58.6 (54.7, 62.4)	<0.001
Cardiovascular health				<0.001
Low	10.1 (9.0, 11.3)	9.2 (8.1, 10.5)	16.2 (13.0, 20.1)	
Moderate	66.3 (63.2, 69.2)	66.2 (62.1, 68.6)	72.0 (67.3, 76.3)	
High	23.6 (20.2, 27.5)	25.9 (21.9, 29.1)	11.7 (7.2, 18.6)	

Variables were presented as weighted percentages or means (95% confidence intervals). Low CVH was defined as a LE8 score of 0 to 49, moderate CVH of 50–79, and high CVH of 80–100.

Abbreviations: DASH, Dietary Approaches to Stop Hypertension; LE8, life’s essential 8; CVH, cardiovascular health.

### The association between LE8 score and gallstones

Tables 2 and 3 show the association of LE8 and its eight sub-scores with gallstones. The results of all models showed that the higher the LE8 score, the lower the likelihood of gallstones. In the fully adjusted model, for every 10-unit increase in LE8 score, the prevalence of gallstones decreased by 17% (OR = 0.83; 95% CI, 0.75–0.91). Convert continuous LE8 scores to the categorical variable CVH as define, in the model adjusted for all covariates, the prevalence of gallstones was significantly reduced by 57% in participants with high CVH compared with low CVH (OR = 0.43; 95% CI, 0.26–0.72), whereas there was no significant association between intermediate CVH and gallstones. Among the associations of gallstones with the eight sub-scores, gallstones were only somewhat negatively associated with the blood glucose score and body mass index score, especially the blood glucose score, which showed a 15% decrease in gallstone odds for every 10-unit increase in the score (OR = 0.85; 95% CI, 0.77–0.94), whereas the remaining six sub-scores (DASH diet score, physical activity score, tobacco exposure score, sleep health score, blood lipid score and blood pressure score) did not have a significant effect on gallstones.

**Table 2:** The associations between Life’s Essential 8 sub-scores and gallstones.

	Model 1	Model 2	Model 3
LE8 score components (Per 10 points increase)	OR (95% CI), p value	OR (95% CI), p value	OR (95% CI), p value
Total LE8 score	0.75 (0.70, 0.81) <0.001	0.79 (0.73, 0.86) <0.001	0.83 (0.75, 0.91) 0.02
DASH diet score	1.00 (0.96, 1.05) 0.99	0.97 (0.93, 1.01) 0.15	0.97 (0.95, 1.05) 0.19
Physical activity score	0.95 (0.92, 0.98) 0.002	0.98 (0.95, 1.01) 0.25	0.99 (0.95, 1.03) 0.42
Tobacco exposure score	0.99 (0.95, 1.02) 0.48	0.97 (0.93, 1.01) 0.13	0.96 (0.91, 1.02) 0.14
Sleep health score	0.97 (0.92, 1.03) 0.35	0.97 (0.91, 1.04) 0.37	0.97 (0.90, 1.06) 0.41
Body mass index score	0.89 (0.86, 0.92) <0.001	0.88 (0.85, 0.91) <0.001	0.89 (0.85, 0.95) 0.01
Blood lipid score	0.97 (0.94, 1.01) 0.12	1.00 (0.97, 1.04) 0.87	1.00 (0.95, 1.05) 0.92
Blood glucose score	0.85 (0.79, 0.92) <0.001	0.86 (0.80, 0.93) <0.001	0.85 (0.77, 0.94) 0.03
Blood pressure score	0.91 (0.88, 0.95) <0.001	0.97 (0.93, 1.02) 0.2	0.99 (0.93, 1.05) 0.64

Model 1: no covariates were adjusted.

Model 2: age, gender, race, education level, poverty ratio and marital status were adjusted.

Model3: age, gender, race, education level, poverty ratio, marital status, diabetes, cancer, cardiovascular disease and taking anti-hypertensive or lipid-lowering medicine were adjusted.

Abbreviations: 95% CI: 95% confidence interval; OR: odd ratio.

**Table 3:** The associations between LE8 score and gallstones.

	Model 1	Model 2	Model 3
LE8 score	OR (95% CI)	OR (95% CI)	OR (95% CI)
**Per 10 points increase**	0.75 (0.70, 0.81)	0.79 (0.73, 0.86)	0.83 (0.75, 0.91)
**Cardiovascular health**			
Low (0–49)	Reference	Reference	Reference
Moderate (50–79)	0.63 (0.48, 0.82)	0.72 (0.56, 0.94)	0.85 (0.62, 1.15)
High (80–100)	0.26 (0.17, 0.42)	0.34 (0.21, 0.55)	0.43 (0.26, 0.72)
P for trend	<0.001	<0.001	<0.001

Model 1: no covariates were adjusted.

Model 2: age, gender, race, education level, poverty ratio and marital status were adjusted.

Model3: age, gender, race, education level, poverty ratio, marital status, diabetes, cancer, cardiovascular disease and taking anti-hypertensive or lipid-lowering medicine were adjusted.

Abbreviations: 95% CI: 95% confidence interval; OR: odd ratio.

**Fig 2:**
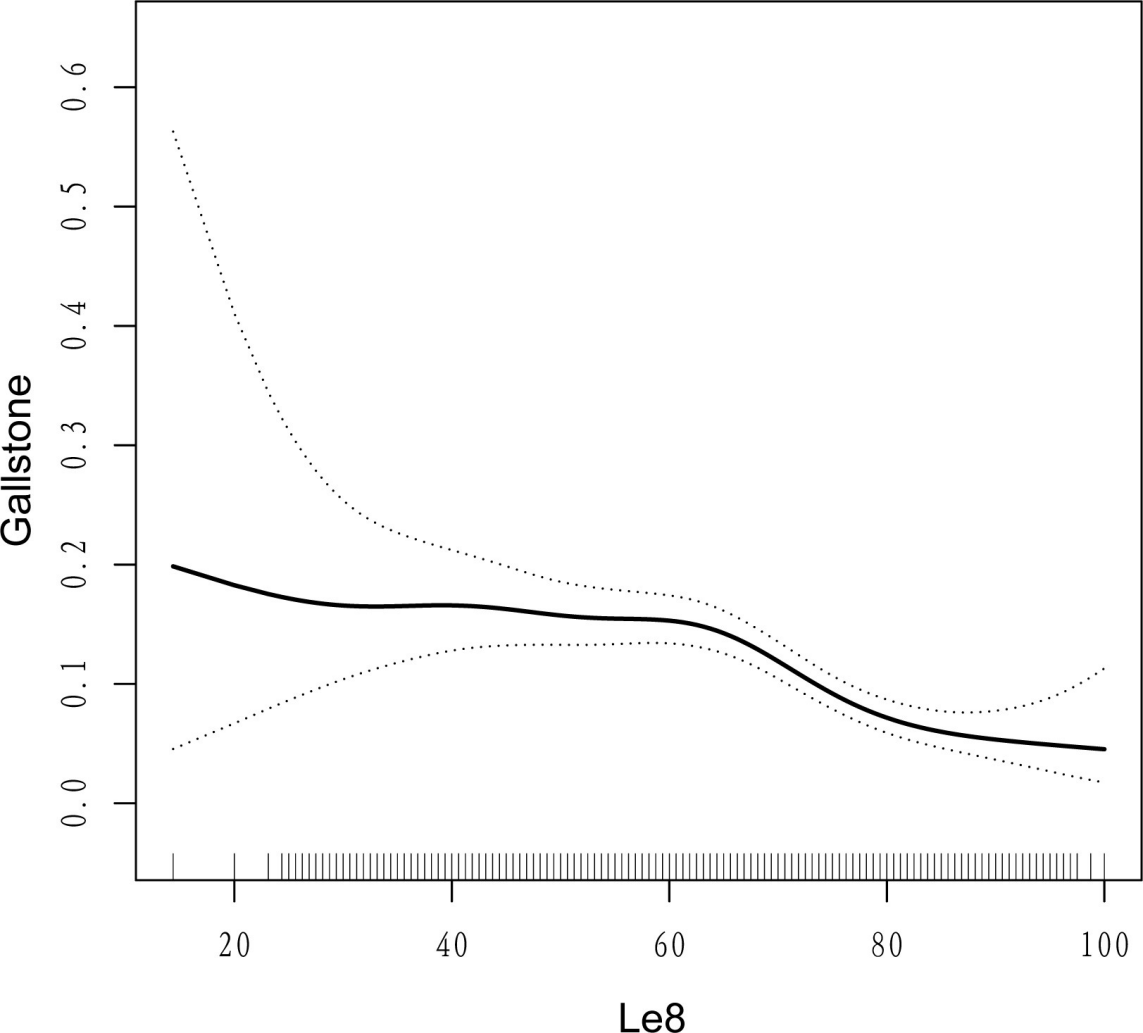
The generalized additive model for the relationship between LE8 score and gallstones.

Based on these results, the nonlinear relationship between LE8 score and gallstones was further investigated using smooth curve fitting and generalized additive models (GAM). We find a nonlinear negative correlation and a potential saturation effect (Fig 2), The threshold effect analysis (Table 4) also revealed that the minimum threshold for the protective effect was 66.25 points (Log likelihood ratio < 0.001). On the right side of the inflection point, the odds of developing gallstones decreased progressively with increasing LE8 score (OR = 0.95; 95%CI, 0.93–0.96), whereas on the left side of it, there was no significant protective effect for both, and it was not statistically significant (OR = 0.99; 95%CI, 0.98–1.01).

**Table 4:** Threshold effect analysis of Life’s Essential 8 score on gallstones using two-piecewise linear regression model.

Gallstones		After adjustment
Model 1	OR(95%CI)	0.98 (0.97, 0.99)
	p for trend	<0.001
Model 2	Inflection point(K)	66.25
	LE8 score < 66.25	0.99 (0.98, 1.01)
	LE8 score > 66.25	0.95 (0.93, 0.96)
	Logarithmic likelihood ratio test P value	<0.001

Model 1: Standard linear model.

Model 2: Two-piecewise linear model.

Adjusted for age, gender, race, education level, poverty ratio, marital status, diabetes, cancer, cardiovascular disease and taking anti-hypertensive or lipid-lowering medicine.

### Subgroups analysis

Subgroup analyses and interaction tests assessed the potential association between LE8 score and gallstones in different populations. The results presented in Table 5 demonstrate significant interactions between the association and age, as well as the use of blood pressure and lipid-lowering drugs, across all subgroups (p for interaction < 0.05), The protective effect of LE8 score on gallstones was stronger in those under 60 years old(OR = 0.72; 95%CI, 0.65–0.81) and not taking medication (OR = 0.71; 95%CI, 0.64–0.80), whereas a statistically insignificant decrease in gallstone odds was observed in the opposite population, although a decrease in gallstone odds was also observed. In addition, the negative association between LE8 score and gallstones remained robust across gender, race, poverty ratio, diabetes, cardiovascular disease, and cancer populations (all p for interaction > 0.05).

**Table 5:** Subgroup analysis.

	Gallstones OR (95% CI)	P for interaction
Age		0.007
20–59	0.72 (0.65, 0.81)	
≥ 60	0.90 (0.80, 1.03)	
Gender		0.257
Male	0.84 (0.73, 0.97)	
Female	0.76 (0.69, 0.84)	
Race		0.472
Mexican American	0.69 (0.54, 0.88)	
Other Hispanic	0.87 (0.65, 1.17)	
Non-Hispanic White	0.83 (0.73, 0.94)	
Non-Hispanic Black	0.84 (0.68, 1.03)	
Other Races	0.71 (0.57, 0.88)	
Poverty ratio		0.901
< 1.3	0.82 (0.69, 0.97)	
1.3–3.5	0.76 (0.67, 0.87)	
> 3.5	0.81 (0.68, 0.95)	
Unclear	0.77 (0.59, 0.99)	
Diabetes		0.733
Yes	0.81 (0.68, 0.97)	
No	0.78 (0.71, 0.86)	
Cancer		0.629
Yes	0.76 (0.62, 0.92)	
No	0.80 (0.73, 0.87)	
Cardiovascular disease		0.640
Yes	0.74 (0.55, 0.99)	
No	0.79 (0.72, 0.86)	
Taking anti-hypertensive or lipid-lowering medicine		0.006
Yes	0.89 (0.79, 1.00)	
No	0.71 (0.64, 0.80)	

The results of subgroup analysis were adjusted for all covariates except effect modifier.

OR, odds ratio; 95% CI, 95% confidence interval.

Besides, considering that parity status may be an important confounding factor, we performed an additional subgroup analysis, adjusting the parity status as a covariate in the female population, to test the robustness of our results. Our findings were generally consistent in the new analysis, including the baseline characteristics of the 1,427-woman group after adjusting for parity status were similar to those of the general population (S2 Table), LE8 score was negatively correlated with gallstones (S3 and S4 Tables), a saturation effect was detected by smoothed curve fitting, and both demonstrated a significant protective effect of high CVH (S5 Table and S1 Fig), and subgroup analyses showed a stronger protective effect of LE8 score in those under 60 years of age, but there was no significant interaction in those whether or not they were on medication, which may be due to the relatively small sample size of the female group (S6 Table).

## Discussion

The results of this cross-sectional study with 3570 participants showed a negative correlation between gallstones and LE8 score. There was also a saturation effect, with a minimum threshold of 66.25 for the LE8 score to exert a protective effect against gallstones. In subgroup analyses, the inverse association between LE8 score and gallstones was found to be more pronounced among individuals under 60 years old and those not taking lipid-lowering or blood-pressure medications. Our results suggest that earlier and more rigorous interventions based on health behaviors and health factors that are components of the LE8 score may be beneficial in reducing the odds of gallstones.

To the best of our knowledge, this study is the first to evaluate the relationship between gallstones and CVH using LE8 score, and previous studies have reported an association between gallstones and some cardiovascular risk factors. A study by Yuan et al. found that obesity and type 2 diabetes were independent risk factors for gallstone development [20]; A large meta-analysis noted that smokers had a 19% increased relative risk of developing gallstones, and that the risk rose as one smoked more cigarettes [21]; In a large prospective study involving 500,000 people in China, Pang et al. reported that high physical activity was negatively associated with the risk of hepatobiliary diseases [22]; Wirth et al. found in a prospective study involving 43,635 U.S. individuals that increased adherence to a variety of healthy eating patterns, including Dietary Approaches to Stop Hypertension (DASH), reduced the incidence of gallstones [23].

Despite the lack of clarity on the mechanisms behind the association between gallstones and LE8 score, a large number of studies have revealed that metabolic syndrome and lifestyle contribute significantly to the pathophysiology of gallstones, and our findings are consistent with previous studies demonstrating the association between metabolic syndrome and gallstones [24–26]. First, insulin resistance during metabolic syndrome dysregulates the hepatocyte transcription factor forkhead box protein O1 (FOXO1), which induces ABCG5 and ABCG8 to promote cholesterol secretion from bile [27], resulting in supersaturation of cholesterol and stone formation; Excess cholesterol can also be taken up by epithelial cells lining the gallbladder wall and converted to cholesteryl esters for storage in the mucosa and lamina propria, which stiffens the myometrium of smooth muscle cells, disrupts the cholecystokinin 1 receptor signaling cascade, and causes uncoupling of G-protein-mediated signaling, suchas Gq/11alpha, Giα1–2 and Giα3 [28], to the point of dysfunction of gallbladder dynamics and impaired bile emptying. Secondly, the production of very low-density lipoproteins is increased in insulin-resistant status [29]. The flow of their residues to the liver increases so that the availability of hepatic cholesterol for secretion into the bile is increased [8], which favors supersaturation and stone formation. At the same time, disturbances of lipid metabolism are also a common etiological link between gallstones and cardiovascular disease [30]. Finally, elevated pro-inflammatory cytokines associated with insulin resistance impair the contractility of the gallbladder wall [31], prolonging the retention time of supersaturated bile in the lumen of the gallbladder and potentially facilitating cholesterol crystallization and crystal growth, resulting in gallstones formation [32].

There are several other interesting findings in this study; firstly we observed a saturation effect between LE8 and gallstones, where both remained stable in the lower range of LE8 score, and the prevalence of gallstones tended to decrease at higher levels, and a minimum threshold for a beneficial association was identified, which suggests that adherence to higher LE8 scores has a more protective effect against gallstones. Second, in subgroup analyses, we found that the LE8 score showed a stronger protective effect in younger people, whereas there was no statistically significant effect in people over 60 years old. Current research has confirmed that cholesterol oversaturation in bile is one of the three main mechanisms in the pathogenesis of gallstones [32], that excessive cholesterol secretion is the leading cause of cholesterol oversaturation [33] and the most important cause of lithogenic bile [34], and that cholesterol secretion increases with age [35], We thus speculate that age may be a more influential risk factor for gallstones in individuals over 60 years old, and that reducing the risk of metabolism-related factors does not offset the effect of growing age on gallstone formation. This suggests that we should assess and manage our cardiovascular health more at a younger age to minimize the occurrence of gallstones effectively. In addition, similar results were seen in the subgroup of people taking antihypertensive or lipid-lowering medications, suggesting that a high CVH is more beneficial in terms of decreasing the prevalence of gallstones in the drug-naive population. Lipid-lowering drugs have been proven to reduce the odds of gallstones [36], so the protective effect of CVH on gallstones was not significant in this population. Finally, in the logistic regression of LE8 sub-scores with gallstones, we found that gallstones were negatively correlated only with blood glucose and body mass index scores. It has been suggested that an assessment of overall cardiovascular health status may have a greater impact on an individual’s health than the sum of its parts [37] with possible synergistic effects [15]. Studies have also shown that the prevalence of gallstones increases significantly with the increase of metabolic risk factors [38]. Given that the health behaviors and health factors encompassed by LE8 are interrelated, such that improvements in blood pressure and glycemic status are associated with healthy dietary patterns and smoking cessation [39], the potential pathways by which they influence gallstone pathogenesis are likely to be intertwined. Therefore, rather than improving individual risk factors, an integrated management approach represented by the LE8 score may be more effective and efficient in preventing gallstones, which is comprehensive and easy to apply, and promotes adherence to desirable health behaviors and health factors, rather than just starting treatment when adverse risks arise.

Our study has several strengths. First, the data for this study were derived from NHANES based on national population sampling, and the appropriate weights were used to make the results more representative. In addition, we used the updated LE8 score to assess CVH, identified a saturation effect between CVH and gallstones, and determined the minimum threshold for a beneficial association. However, potential limitations of this study should also be considered. First, the assessment of gallstones and partial sub-scores was obtained from self-report questionnaires, which may introduce recall bias leading to estimation errors; Second, despite adjusting for some potential covariates, the effects of other possible confounders cannot be completely ruled out; Finally, due to the limitations of cross-sectional studies, our study was unable to draw a definitive causal relationship, more prospective studies are still needed to validate the rigor of the results of this study.

## Conclusion

This study suggests a nonlinear negative correlation between LE8 score and gallstones, and that those under 60 years old and those not taking antihypertensive or lipid-lowering medications may benefit more from this. Given the inherent limitations of cross-sectional studies, further research are essential to confirm the causality of this association and to elucidate the underlying mechanisms.

## Supporting information

S1 Fig The generalized additive model for the relationship between LE8 score and gallstones in female. (TIF)

S1 Table Definition and scoring approach for the American Heart Association’s Life’s Essential 8 score. (DOCX)

S2 Table Basic characteristics of female participants (n = 1429) in the NHANES 2017–2018. (DOCX)

S3 Table The associations between the Life’s Essential 8 scores and gallstone in female. (DOCX)

S4 Table Sensitivity analysis of the association of the LE8 scores with gallstone in female. (DOCX)

S5 Table Threshold effect analysis of Life’s Essential 8 scores on Gallstone using two-piecewise linear regression model. (DOCX)

S6 Table Subgroup analysis. (DOCX)
