# Supplementary material for: Associations between Life’s Essential 8 and gallstones among US adults: A cross-sectional study from NHANES 2017–2018
Source: PLoS One. 2024 Oct 30;19(10):e0312857. doi: 10.1371/journal.pone.0312857 (PMC11524467; doi:10.1371/journal.pone.0312857)
Supplement: S1 Table — (DOCX) [file pone.0312857.s002.docx]

**S1 Table. Definition and scoring approach for the American Heart Association’s Life’s Essential 8 score.**

| **Domain** | **CVH Metric** | **Measurement** | **Quantification and Scoring of CVH Metric** |
| --- | --- | --- | --- |
| **Health Behaviors** | **Diet** | **Measurement:** Self-reported daily intake of a DASH-style eating pattern  **Example tools for**  **measurement:** DASH diet score (populations) | Quantiles of DASH-style diet adherence  **Scoring (Population):**  Points Quantile  100 ≥95^th^ percentile (top/ideal diet)  80 75^th^ – 94^th^ percentile  50 50^th^ – 74^th^ percentile  25 25^th^ – 49^th^ percentile  0 1^st^ – 24^th^ percentile (bottom/least ideal quartile) |
|  | **Physical activity** | **Measurement:** Self-reported minutes of moderate or vigorous physical activity per week  **Example tools for measurement:** NHANES PAQ-K questionnaire | **Metric:** Minutes of moderate (or greater) intensity activity per week  **Scoring:**  Points Minutes  100 ≥150  90 120 – 149  80 90 – 119  60 60 – 89  40 30 – 59  20 1 – 29  0 0 |
|  | **Nicotine exposure** | **Measurement:** Self-reported use of cigarettes or inhaled nicotine- delivery system  **Example tools for measurement:** NHANES SMQ | **Metric:** Combustible tobacco use and/or inhaled NDS use; or secondhand smoke exposure  **Scoring:**  Points Status  100 Never smoker  75 Former smoker, quit ≥5 yrs  50 Former smoker, quit 1 - <5 yrs  25 Former smoker, quit <1 year, or currently using inhaled NDS  0 Current smoker  Subtract 20 points (unless score is 0) for living with active indoor smoker in home |
|  | **Sleep health** | **Measurement:** Self-reported average hours of sleep per night  **Example tools for measurement:** “On average, how many hours of sleep do you get per night?” Consider objective sleep/actigraphy data from wearable technology, if available | **Metric:** Average hours of sleep per night  **Scoring:**  Points Level  100 7 – <9  90 9 – <10  70 6 – <7  40 5 – <6 or ≥10  20 4 – <5  0 <4 |
| **Health Factors** | **Body mass index** | **Measurement:** Body weight (kg) divided by height squared (m^2^)  **Example tools for measurement:** Objective measurement of height and weight | **Metric:** Body mass index (kg/m^2^)  **Scoring:** Points Level 100 <25  70 25.0 – 29.9  30 30.0 – 34.9  15 35.0 – 39.9  0 ≥40.0 |
|  | **Blood lipids** | **Measurement:** Plasma total and HDL-cholesterol with calculation of non-HDL-cholesterol  **Example tools for measurement:** Fasting or non-fasting blood sample | **Metric:** Non-HDL-cholesterol (mg/dL)  **Scoring:**  Points Level  100 <130  60 130 – 159  40 160 – 189  20 190 – 219  0 ≥220  If drug-treated level, subtract 20 points |
|  | **Blood glucose** | **Measurement:** Fasting blood glucose or casual hemoglobin A1c  **Example tools for measurement:**  Fasting (FBG, HbA1c) or nonfasting (HbA1c) blood sample | **Metric:** Fasting blood glucose (mg/dL) or Hemoglobin A1c (%)  **Scoring:**  Points Level  100 No history of diabetes and FBG <100 (or HbA1c < 5.7)  60 No diabetes and FBG 100 – 125 (or HbA1c 5.7-6.4) (Pre-diabetes)  40 Diabetes with HbA1c <7.0  30 Diabetes with HbA1c 7.0 – 7.9  20 Diabetes with HbA1c 8.0 – 8.9  10 Diabetes with Hb A1c 9.0 – 9.9  0 Diabetes with HbA1c ≥10.0 |
|  | **Blood pressure** | **Measurement:** Appropriately measured systolic and diastolic blood pressure  **Example tools for measurement:** Appropriately sized blood pressure cuff | **Metric:** Systolic and diastolic blood pressure (mm Hg)  **Scoring:**  Points Level  100 <120/<80 (Optimal)  75 120-129/<80 (Elevated)  50 130-139 or 80-89 (Stage I HTN)  25 140-159 or 90-99  0 ≥160 or ≥100  Subtract 20 points if treated level |
