# Supplementary material for: Associations between Life’s Essential 8 and gallstones among US adults: A cross-sectional study from NHANES 2017–2018
Source: PLoS One. 2024 Oct 30;19(10):e0312857. doi: 10.1371/journal.pone.0312857 (PMC11524467; doi:10.1371/journal.pone.0312857)
Supplement: S2 Table — (DOCX) [file pone.0312857.s003.docx]

**S2 Table. Basic characteristics of female participants (n = 1429) in the NHANES 2017–2018.**

|  | **Overall** | **Non-GALLSTONE** | **GALLSTONE** | ***P*-value** |
| --- | --- | --- | --- | --- |
|  | **N=1429** | **N=1188** | **N=241** |  |
| Age(years) | 52.7 (51.6, 53.8) | 51.7 (50.7, 52.8) | 57.2 (55.9, 58.5) | <0.001 |
| Parity status | 2.44 (2.3, 2.6) | 2.4 (2.3, 2.5) | 2.5 (2.3, 2.7) | 0.400 |
| Age strata |  |  |  | <0.001 |
| 20-59 | 63.6 (59.5, 67.6) | 66.3 (62.2, 70.1) | 51.6 (46.3, 56.8) |  |
| ≥ 60 | 36.4 (32.4, 40.5) | 33.7 (30.0, 37.8) | 48.4 (43.2, 53.7) |  |
| Race(%) |  |  |  | 0.451 |
| Mexican American | 7.1 (5.0, 9.9) | 7.1 (4.8, 10.3) | 7.0 (4.7, 10.1) |  |
| Other Hispanic | 6.2 (4.5, 8.4) | 6.6 (4.5, 9.6) | 4.4 (1.6, 11.8) |  |
| Non-Hispanic White | 66.8 (59.8, 73.0) | 65.9 (57.6, 73.3) | 70.6 (61.1, 78.6) |  |
| Non-Hispanic Black | 11.4 (8.1, 15.7) | 12.3 (8.6, 17.3) | 7.3 (5.1, 10.2) |  |
| Other Races | 8.6 (5.9, 12.3) | 8.1 (5.4, 12.0) | 10.7 (4.4, 23.9) |  |
| Poverty ratio |  |  |  | 0.545 |
| < 1.3 | 19.9 (17.8, 22.2) | 19.5 (16.9, 22.3) | 21.4 (14.3, 30.8) |  |
| 1.3–3.5 | 31.8 (27.8, 36.0) | 31.0 (27.4, 35.0) | 35.3 (25.7, 46.3) |  |
| > 3.5 | 39.7 (34.8, 44.8) | 40.2 (35.1, 45.6) | 37.3 (28.2, 47.6) |  |
| Unclear | 8.6 (7.4, 10.1) | 8.4 (6.7, 10.5) | 5.9 (2.8, 12.0) |  |
| Marital status |  |  |  | 0.697 |
| Coupled | 65.3 (62.3, 68.2) | 65.5 (62.0, 68.9) | 64.3 (55.1, 72.6) |  |
| Widowed or separated | 26.8 (24.6, 29.2) | 26.4 (23.7, 29.2) | 28.9 (21.7, 36.8) |  |
| Never married | 7.9 (6.0, 10.3) | 8.1 (6.1, 10.8) | 6.8 (4.3, 11.2) |  |
| Education level (%) |  |  |  | 0.373 |
| Less than high school | 10.1 (8.3, 12.1) | 9.9 (7.9, 12.3) | 10.9 (7.5, 15.6) |  |
| High school or GED | 27.4 (24.7, 30.4) | 26.5 (22.8, 30.5) | 31.8 (23.3, 41.7) |  |
| Above high school | 62.5 (59.1, 65.8) | 63.6 (59.1, 68.0) | 57.3 (49.2, 65.0) |  |
| Diabetes (%) |  |  |  | 0.001 |
| Yes | 14.3 (11.5, 17.6) | 12.0 (9.3, 15.4) | 24.7 (16.6, 35.2) |  |
| No | 85.7 (82.4, 88.5) | 88.0 (84.6, 90.7) | 75.3 (64.9, 83.4) |  |
| Cancer (%) |  |  |  | 0.374 |
| Yes | 14.0 (11.5, 17.4) | 13.4 (10.3, 17.3) | 16.6 (10.6, 25.0) |  |
| No | 86.0 (82.4, 88.5) | 86.6 (82.7, 89.7) | 83.4 (75.0, 89.4) |  |
| Cardiovascular disease (%) |  |  |  | 0.695 |
| Yes | 2.9 (1.6, 5.3) | 2.8 (1.3, 5.9) | 3.4 (1,8, 6.3) |  |
| No | 97.1 (94.7, 98.4) | 97.2 (94.1, 98.7) | 96.6 (93.7, 98.2) |  |
| Taking anti-hypertensive or lipid-lowering medicine (%) |  |  |  | <0.001 |
| Yes | 37.3 (33.4, 41.3) | 34.4 (31.1, 37.8) | 50.6 (39.7, 61.4) |  |
| No | 62.7 (58.7, 66.6) | 65.6 (62.2, 68.9) | 49.4 (38.6, 60.3) |  |
| LE8 scores (out of 100 possible points |  |  |  |  |
| LE8 score | 67.5 (66.1, 68.9) | 68.7 (67.3, 70.2) | 61.8 (60.5, 63.0) | <0.001 |
| DASH diet score | 39.2 (36.1, 42.4) | 39.1 (35.9, 42.3) | 39.9 (34.8, 44.9) | 0.735 |
| Physical activity score | 69.6 (65.9, 73.3) | 70.3 (66.3, 74.3) | 66.4 (60.0, 72.8) | 0.270 |
| Tobacco exposure score | 76.4 (73.9, 79.0) | 77.8 (74.3, 81.3) | 70.1 (65.0, 75.2) | 0.069 |
| Sleep health score | 84.4 (82.4, 86.3) | 84.7 (82.3, 87.0) | 83.0 (78.8, 87.1) | 0.525 |
| Body mass index score | 54.3 (50.4, 58.1) | 57.5 (53.7, 61.3) | 39.4 (33.1, 45.8) | <0.001 |
| Blood lipids score | 66.0 (63.1, 68.8) | 67.0 (64.1, 69.9) | 61.3 (56.1, 66.4) | 0.040 |
| Blood glucose score | 83.2 (81.4, 84.9) | 84.7 (82.9, 86.5) | 76.1 (72.3, 79.8) | <0.001 |
| Blood pressure score | 66.9 (64.4, 69.4) | 68.8 (66.4, 71.2) | 58.1 (54.1, 62.0) | <0.001 |
| Cardiovascular health |  |  |  | <0.001 |
| Low | 11.0 (8.6, 13.9) | 9.7 (7.2, 12.9) | 17.0 (12.7, 22.4) |  |
| Moderate | 67.1 (63.9, 70.2) | 66.0 (62.4, 69.5) | 72.2 (63.4, 79.5) |  |
| High | 21.9 (18.0, 26.4) | 24.3 (20.1, 29.0) | 10.8 (6.0, 18.8) |  |

Variables were presented as weighted percentages or means (95% confidence intervals).

DASH, Dietary Approaches to Stop Hypertension; LE8, life’s essential 8; CVH, cardiovascular health.

Low CVH was defined as a LE8 score of 0 to 49, moderate CVH of 50–79, and high CVH of 80–100.
