# Supplementary material for: Associations between Life’s Essential 8 and gallstones among US adults: A cross-sectional study from NHANES 2017–2018
Source: PLoS One. 2024 Oct 30;19(10):e0312857. doi: 10.1371/journal.pone.0312857 (PMC11524467; doi:10.1371/journal.pone.0312857)
Supplement: S3 Table — (DOCX) [file pone.0312857.s004.docx]

**S3 Table. The associations between the Life’s Essential 8 scores and gallstone in female.**

|  | **Model 1** | **Model 2** | **Model 3** |
| --- | --- | --- | --- |
| **LE8 score components (Per 10 points increase)** | **OR (95% CI), p value** | **OR (95% CI), p value** | **OR (95% CI), p value** |
| Total LE8 score | 0.71 (0.65, 0.78)  <0.001 | 0.72 (0.67, 0.79)  <0.001 | 0.75 (0.65, 0.87)  <0.001 |
| DASH diet score | 1.01 (0.96, 1.06)  0.73 | 0.99 (0.99, 1.05)  0.85 | 1.00 (0.95, 1.05)  0.96 |
| Physical activity score | 0.98 (0.94, 1.02)  0.26 | 0.99 (0.95, 1.04)  0.73 | 0.99 (0.95, 1.04)  0.75 |
| Tobacco exposure score | 0.95 (0.90, 1.00)  0.07 | 0.94 (0.89, 0.99)  0.02 | 0.94 (0.89, 0.99)  0.02 |
| Sleep health score | 0.97 (0.89, 1.06)  0.52 | 0.96 (0.88, 1.05)  0.54 | 0.96 (0.88, 1.05)  0.37 |
| Body mass index score | 0.86 (0.81, 0.92)  <0.001 | 0.85 (0.79, 0.90)  <0.001 | 0.86 (0.79, 0.93)  <0.001 |
| Blood lipid score | 0.94 (0.88, 0.99)  0.03 | 0.97 (0.92, 1.02)  0.45 | 0.97 (0.92, 1.02)  0.20 |
| Blood glucose score | 0.88 (0.83, 0.94)  <0.001 | 0.91 (0.84, 0.97)  0.01 | 0.97 (0.85, 1.11)  0.63 |
| Blood pressure score | 0.91 (0.88, 0.94)  <0.001 | 0.94 (0.90, 0.99)  0.03 | 0.96 (0.92, 1.01)  0.14 |

Model 1: no covariates were adjusted.

Model 2: age, gender, race, education level, poverty ratio, marital status and parity status were adjusted.

Model3: age, gender, race, education level, poverty ratio, marital status, parity status, diabetes, cancer, cardiovascular disease and taking anti-hypertensive or lipid-lowering medicine were adjusted.

95% CI :95% confidence interval.

OR: odd ratio.
