# Supplementary material for: Associations between Life’s Essential 8 and gallstones among US adults: A cross-sectional study from NHANES 2017–2018
Source: PLoS One. 2024 Oct 30;19(10):e0312857. doi: 10.1371/journal.pone.0312857 (PMC11524467; doi:10.1371/journal.pone.0312857)
Supplement: S4 Table — (DOCX) [file pone.0312857.s005.docx]

**S4 Table. Sensitivity analysis of the association of the LE8 scores with gallstone in female.**

|  | **Model 1** | **Model 2** | **Model 3** |
| --- | --- | --- | --- |
| **LE8 score** | **OR (95% CI), p value** | **OR (95% CI), p value** | **OR (95% CI), p value** |
| **Per 10 points increase** | 0.71 (0.65, 0.78) | 0.72 (0.67, 0.79) | 0.75 (0.65, 0.87) |
| **Cardiovascular health** |  |  |  |
| Low (0-49) | Reference | Reference | Reference |
| Moderate (50-79) | 0.62 (0.39, 0.99) | 0.64 (0.41, 1.02) | 0.72 (0.40, 1.32) |
| High (80-100) | 0.25 (0.14, 0.44) | 0.29 (0.18, 0.49) | 0.37 (0.18, 0.73) |
| P for trend | 0.029 | 0.303 | 0.131 |

Model 1: no covariates were adjusted.

Model 2: age, gender, race, education level, poverty ratio, marital status and parity status were adjusted.

Model3: age, gender, race, education level, poverty ratio, marital status, parity status, diabetes, cancer, cardiovascular disease and taking anti-hypertensive or lipid-lowering medicine were adjusted.

95% CI :95% confidence interval.

OR: odd ratio.
