# Supplementary material for: Associations between Life’s Essential 8 and gallstones among US adults: A cross-sectional study from NHANES 2017–2018
Source: PLoS One. 2024 Oct 30;19(10):e0312857. doi: 10.1371/journal.pone.0312857 (PMC11524467; doi:10.1371/journal.pone.0312857)
Supplement: S5 Table — (DOCX) [file pone.0312857.s006.docx]

**S5 Table. Threshold effect analysis of Life’s Essential 8 scores on Gallstone using two-piecewise linear regression model.**

| **Gallstones** |  | **After adjustment** |
| --- | --- | --- |
| Model 1 | OR(95%CI) | 0.98 (0.96, 0.99) |
|  | p for trend | <0.001 |
| Model 2 | Inflection point(K) | 60.00 |
|  | LE8 score < 60.00 | 0.99 (0.97, 1.02) |
|  | LE8 score > 60.00 | 0.97 (0.93, 1.00) |
|  | Logarithmic likelihood ratio test P value | 0.062 |

Model 1: Standard linear model.

Model 2: Two-piecewise linear model.

Adjusted for age, parity status, race, education level, poverty ratio, marital status, diabetes, cancer, cardiovascular disease and taking anti-hypertensive or lipid-lowering medicine.
