# Supplementary material for: Associations between Life’s Essential 8 and gallstones among US adults: A cross-sectional study from NHANES 2017–2018
Source: PLoS One. 2024 Oct 30;19(10):e0312857. doi: 10.1371/journal.pone.0312857 (PMC11524467; doi:10.1371/journal.pone.0312857)
Supplement: S6 Table — (DOCX) [file pone.0312857.s007.docx]

**S6 Table. Subgroup analysis.**

|  | **Gallstones**  **OR (95% CI**) | **P for interaction** |
| --- | --- | --- |
| Age |  | 0.006 |
| 20-59 | 0.61 (0.50, 0.74) |  |
| ≥ 60 | 0.94 (0.70, 1.25) |  |
| Parity status |  | 0.495 |
| 0-3 | 0.76 (0.63, 0.92) |  |
| 4-6  > 6 | 0.61 (0.43, 0.88)  0.54 (0.37, 0.76) |  |
| Race |  | 0.572 |
| Mexican American | 0.94 (0.60, 1.47) |  |
| Other Hispanic | 1.16 (0.63, 2.14) |  |
| Non-Hispanic White | 0.73 (0.58, 0.93) |  |
| Non-Hispanic Black | 0.84 (0.61, 1.16) |  |
| Other Races | 0.56 (0.33, 0.94) |  |
| Poverty ratio |  | 0.413 |
| < 1.3 | 0.80 (0.63, 1.02) |  |
| 1.3–3.5 | 0.69 (0.54, 0.89) |  |
| > 3.5 | 0.81 (0.57, 1.14) |  |
| Unclear | 0.35 (0.18, 0.66) |  |
| Diabetes |  | 0.218 |
| Yes | 0.88 (0.59, 1.30) |  |
| No | 0.73 (0.59, 0.89) |  |
| Cancer |  | 0.313 |
| Yes | 0.67 (0.50, 0.89) |  |
| No | 0.75 (0.63, 0.89) |  |
| Cardiovascular disease |  | 0.091 |
| Yes | 0.54 (0.38, 0.78) |  |
| No | 0.75 (0.65, 0.87) |  |
| Taking anti-hypertensive or lipid-lowering medicine |  | 0.356 |
| Yes | 0.83 (0.66, 1.04) |  |
| No | 0.70 (0.55, 0.87) |  |

The results of subgroup analysis were adjusted for all covariates except effect modifier.

OR, odds ratio; 95% CI, 95% confidence interval.
