# Supplementary material for: Associations between Life’s Essential 8 and gallstones among US adults: A cross-sectional study from NHANES 2017–2018
Source: PLoS One. 2024 Oct 30;19(10):e0312857. doi: 10.1371/journal.pone.0312857 (PMC11524467; doi:10.1371/journal.pone.0312857)
Supplement: Attachment — Submitted filename: Response to Reviewers.docx [file pone.0312857.s008.docx]

Dear Editor and Reviewers,

Thank you for your giving us an opportunity to revise our manuscript. On behalf of all the contributing authors, I would like to express our sincere appreciation of your letter and reviewers’ constructive comments concerning our manuscript entitled “Associations between Life’s essential 8 and gallstones among US adults: a cross-sectional study from NHANES 2017-2018” (PONE-D-24-20718). These opinions help to improve the academic rigor of our article. We have studied these comments carefully and made modifications which we hope meet with your approval.

Revised portions are marked red in the manuscript. All comments are laid out below in italicized font and specific concerns have been numbered. Point-by-point responses to all nice reviewers are listed below in blue.

All authors have read and approved the re-submission of the manuscript. If you have any questions, please let me know.

Thank you for your consideration of our paper and we are looking forward to hearing from you.

Sincerely yours,

Lanyu Chen

chenlanyu@163.com

--------------------------

**Response to Reviewer 1**

**Reviewer 1**:

*The manuscript provides valuable insights into the relationship between cardiovascular health and gallstone prevalence. Addressing the following points could enhance the study's validity and make a significant contribution to the field:*

**Comment 1**. *The cross-sectional design of the study limits causal inference. The observed associations do not confirm that improvements in LE8 scores lead to reduced gallstone prevalence. Therefore, be cautious in the conclusions and choice of language used.*

**Response 1**. Thank you very much for your professional advice. It was our oversight to overstate the relationship between LE8 scores and gallstones in certain sections of the manuscript. We have reviewed numerous NHANES-based articles and made the corrections (line 43-44 & 347-351). We sincerely hope this revised manuscript will meet your requirements.

**Comment 2**. *The reliance on self-reported data for diagnosing gallstones may introduce misclassification bias, as gallstones could be underreported or inaccurately diagnosed, impacting the study's internal validity.*

**Response 2**. Thank you very much for your comment. Indeed, as you have noted, the reliance on self-reported data for diagnosing gallstones may introduce bias. However, since 1999, self-reporting has been the sole method for diagnosing gallstones in NHANES, and existing research on gallstones has also relied on this approach (Hepatology. 2023;77(6):1882-1895; Lipids Health Dis. 2024;23(1):203). Furthermore, although the prevalence may be underestimated, the missing data is random and is unlikely to have a substantial impact on the results we have described. We have addressed this limitation in the Discussion section and hope for your understanding.

**Comment 3**. *The robustness of the findings could be improved by exploring additional potential confounders, such as parity status, which is a significant factor.*

**Response 3**. Thank you very much for your insightful feedback. Your comments help us identify some shortcomings in adjusting for confounding factors. After reviewing studies related to LE8 and gallstones (Hepatology. 2023;77(6):1882-1895; J Transl Med. 2022;20(1):616), we incorporate additional confounding factors that need adjustment. Specifically, we adjust for the number of pregnancies in our statistical analysis for female participants. The updated values have been added to the Results section, our conclusions remain largely consistent with those previously reported, with some differences discussed in it (line 237-248). Detailed data we add in schedules and charts to upload (Table S2, Table S3, Table S4, Table S5 , Table S6 and Fig S1).

**Comment 4**. *How do the demographic characteristics of the NHANES sample compare to the broader U.S. population? What are the implications for the generalizability of your findings?*

**Response 4**. Thank you very much for your professional advice. NHANES is a national cross-sectional survey that collects information every two years from residents, non-institutionalized U.S. adults and children. Its complex sampling design ensures that the data are nationally representative. Our analysis has been conducted in strict adherence to established guidelines. While there may still be some differences compared to broader U.S. populations, our findings could provide valuable insights into the relationship between cardiovascular health and gallstone incidence. We have provided the necessary introduction in the Methods section and hope for your understanding.

**Comment 5**. *How might the findings of this study influence clinical guidelines or public health policies concerning gallstone prevention?*

**Response 5**. Thank you very much for your comment. Our results suggest that cardiovascular health may be a potential risk factor for the occurrence of gallstones. Identifying these driving factors could assist governments and health agencies in developing targeted health policies, optimizing the allocation of medical resources, and improving the efficiency and quality of healthcare services. Additionally, recognizing these risk factors can aid in predicting future health trends, thereby supporting policymakers and researchers in formulating long-term health plans and response strategies.

**Comment 6**. *Could you elaborate on the rationale for choosing this topic? Why is it important, especially considering that improvements in cardiovascular health could have more significant effects on life-threatening conditions such as coronary artery or cerebrovascular diseases?*

**Response 6**. Thank you very much for your comment. Please allow us to clarify the significance of our research. As you mentioned, cardiovascular health is indeed more prominently associated with life-threatening conditions such as coronary artery disease and cerebrovascular diseases, yet, research in these areas is already quite extensive. Gallstones are also commonly believed to be related to cardiovascular risk factors, as the formation of gallstones and atherosclerosis may share critical pathways, but this knowledge remains limited. We aim to provide our own insights into this relationship. Moreover, symptoms such as biliary colic, angina pectoris, and gallbladder-heart syndrome are critical to differentiate clinically. We hope to draw attention to the relationship between these conditions for clinicians. The LE8, as a novel metric for assessing cardiovascular health, is believed to have the potential to predict various diseases. Thus, we conducted this study with the hope of offering new perspectives on the relationship between cardiovascular health and gallstone incidence. We sincerely hope for your understanding.

**Comment 7**. *Given the potential recall bias associated with self-reported data, did you consider validating the gallstone diagnoses with clinical records, or was this not feasible due to the limitations of the dataset?*

**Response 7**. Thank you very much for your professional comment, and we fully agree with your viewpoints. We acknowledge that relying on self-reported data to diagnose gallstones may introduce bias. However, as mentioned earlier, the limitations of the database have constrained the use of more precise methods. The specific limitations have been detailed in the Discussion section. Our team is currently working on collecting more accurate data, and we hope to address these limitations in the future.

**Comment 8**. *Please provide p-values for each factor in Table 2 to better understand the statistical significance of the findings.*

**Response 8**. We sincerely apologize for the lack of rigor. All P-values have been added in the revised manuscript.

**Comment 9**. *Did you check the data for normal distribution, and which tests were used for the univariate analyses presented in Table 1?*

**Response 9**. Thank you very much for your professional comment. First, we apologize for not specifying the testing methods used. We have now provided additional details in the Methods section (line 133-135). Secondly, we conducted a normality test and found that LE8 does not follow a normal distribution. However, we continue to present it using means and standard deviations for the following reasons:

1) Due to the unique characteristics of complex sampling, the methods and significance of normality testing are still debated. Most studies use means and standard deviations directly when the sample size is sufficiently large.

2) For large samples with complex sampling, even minor variations can affect data normality. To address this, we performed a distribution curve fitting, and the curve closely approximates a normal distribution.

3) LE8 is an index derived from eight parameters through complex calculations, which is not suitable for normality transformation.

Consequently, we have chosen to represent the continuous data using means and confidence intervals (CIs). We sincerely hope for your understanding.

-------------------------------

**Response to Reviewer 2**

**Comment 1**. *Why did the authors not use the latest NHANES version?? The authors could pool the different cycles of NHANES data and analyze them.*

**Response 1**. Thank you for your positive feedback, and we are delighted to receive your recognition. Diagnosis of gallstones was only performed after 2017. However, given the potential impact of the COVID-19 pandemic on cardiovascular health, we have chosen to use only data from 2017-2018. We sincerely hope for your understanding.
