# Supplementary material for: Associations between Life’s Essential 8 and gallstones among US adults: A cross-sectional study from NHANES 2017–2018
Source: PLoS One. 2024 Oct 30;19(10):e0312857. doi: 10.1371/journal.pone.0312857 (PMC11524467; doi:10.1371/journal.pone.0312857)
Supplement: Peer review history

# Peer review: Decision Letter 0

**DOI:** 10.1371/journal.pone.0312857.r001

**Authors:** Mohammad Reza Fattahi

16 Aug 2024

PONE-D-24-20718Associations between Life’s essential 8 and gallstones among US adults: a cross-sectional study from NHANES 2017-2018PLOS ONE

Dear Dr. chen,

Thank you for submitting your manuscript to PLOS ONE. After careful consideration, we feel that it has merit but does not fully meet PLOS ONE’s publication criteria as it currently stands. Therefore, we invite you to submit a revised version of the manuscript that addresses the points raised during the review process.

Please submit your revised manuscript by Sep 30 2024 11:59PM. If you will need more time than this to complete your revisions, please reply to this message or contact the journal office at plosone@plos.org. When you're ready to submit your revision, log on to https://www.editorialmanager.com/pone/ and select the 'Submissions Needing Revision' folder to locate your manuscript file.

Please include the following items when submitting your revised manuscript:

- A rebuttal letter that responds to each point raised by the academic editor and reviewer(s). You should upload this letter as a separate file labeled 'Response to Reviewers'.
- A marked-up copy of your manuscript that highlights changes made to the original version. You should upload this as a separate file labeled 'Revised Manuscript with Track Changes'.
- An unmarked version of your revised paper without tracked changes. You should upload this as a separate file labeled 'Manuscript'.

If you would like to make changes to your financial disclosure, please include your updated statement in your cover letter. Guidelines for resubmitting your figure files are available below the reviewer comments at the end of this letter.

If applicable, we recommend that you deposit your laboratory protocols in protocols.io to enhance the reproducibility of your results. Protocols.io assigns your protocol its own identifier (DOI) so that it can be cited independently in the future. For instructions see: https://journals.plos.org/plosone/s/submission-guidelines#loc-laboratory-protocols. Additionally, PLOS ONE offers an option for publishing peer-reviewed Lab Protocol articles, which describe protocols hosted on protocols.io. Read more information on sharing protocols at https://plos.org/protocols?utm_medium=editorial-email&utm_source=authorletters&utm_campaign=protocols.

We look forward to receiving your revised manuscript.

Kind regards,

Mohammad Reza Fattahi, M.D., M.P.H.

Academic Editor

PLOS ONE

Journal requirements:

When submitting your revision, we need you to address these additional requirements.

1. Please ensure that your manuscript meets PLOS ONE's style requirements, including those for file naming. The PLOS ONE style templates can be found at

https://journals.plos.org/plosone/s/file?id=wjVg/PLOSOne_formatting_sample_main_body.pdf and

https://journals.plos.org/plosone/s/file?id=ba62/PLOSOne_formatting_sample_title_authors_affiliations.pdf

2. Thank you for stating the following financial disclosure:

“Scientific and technological innovation project of China Academy of Chinese Medical Sciences (grant number CI2021A00802)”

Please state what role the funders took in the study.  If the funders had no role, please state: "The funders had no role in study design, data collection and analysis, decision to publish, or preparation of the manuscript."

If this statement is not correct you must amend it as needed.

Please include this amended Role of Funder statement in your cover letter; we will change the online submission form on your behalf.

3. PLOS requires an ORCID iD for the corresponding author in Editorial Manager on papers submitted after December 6th, 2016. Please ensure that you have an ORCID iD and that it is validated in Editorial Manager. To do this, go to ‘Update my Information’ (in the upper left-hand corner of the main menu), and click on the Fetch/Validate link next to the ORCID field. This will take you to the ORCID site and allow you to create a new iD or authenticate a pre-existing iD in Editorial Manager. Please see the following video for instructions on linking an ORCID iD to your Editorial Manager account: https://www.youtube.com/watch?v=_xcclfuvtxQ

4. Please upload a copy of Supporting Information Table (Table S1) which you refer to in your text on page 6.

Additional Editor Comments:

Please Address the reviewer's comments before submitting your revision.

[Note: HTML markup is below. Please do not edit.]

Reviewers' comments:

Reviewer's Responses to Questions

**Comments to the Author**

1. Is the manuscript technically sound, and do the data support the conclusions?

The manuscript must describe a technically sound piece of scientific research with data that supports the conclusions. Experiments must have been conducted rigorously, with appropriate controls, replication, and sample sizes. The conclusions must be drawn appropriately based on the data presented. 

Reviewer #1: Yes

Reviewer #2: Yes

**********

2. Has the statistical analysis been performed appropriately and rigorously? 

Reviewer #1: Yes

Reviewer #2: Yes

**********

3. Have the authors made all data underlying the findings in their manuscript fully available?

The PLOS Data policy requires authors to make all data underlying the findings described in their manuscript fully available without restriction, with rare exception (please refer to the Data Availability Statement in the manuscript PDF file). The data should be provided as part of the manuscript or its supporting information, or deposited to a public repository. For example, in addition to summary statistics, the data points behind means, medians and variance measures should be available. If there are restrictions on publicly sharing data—e.g. participant privacy or use of data from a third party—those must be specified.

Reviewer #1: Yes

Reviewer #2: Yes

**********

4. Is the manuscript presented in an intelligible fashion and written in standard English?

PLOS ONE does not copyedit accepted manuscripts, so the language in submitted articles must be clear, correct, and unambiguous. Any typographical or grammatical errors should be corrected at revision, so please note any specific errors here.

Reviewer #1: Yes

Reviewer #2: Yes

**********

5. Review Comments to the Author

Please use the space provided to explain your answers to the questions above. You may also include additional comments for the author, including concerns about dual publication, research ethics, or publication ethics. (Please upload your review as an attachment if it exceeds 20,000 characters)

Reviewer #1: The manuscript provides valuable insights into the relationship between cardiovascular health and gallstone prevalence. Addressing the following points could enhance the study's validity and make a significant contribution to the field:

1. The cross-sectional design of the study limits causal inference. The observed associations do not confirm that improvements in LE8 scores lead to reduced gallstone prevalence. Therefore, be cautious in the conclusions and choice of language used.

2. The reliance on self-reported data for diagnosing gallstones may introduce misclassification bias, as gallstones could be underreported or inaccurately diagnosed, impacting the study's internal validity.

3. The robustness of the findings could be improved by exploring additional potential confounders, such as parity status, which is a significant factor.

4. How do the demographic characteristics of the NHANES sample compare to the broader U.S. population? What are the implications for the generalizability of your findings?

5. How might the findings of this study influence clinical guidelines or public health policies concerning gallstone prevention?

6. Could you elaborate on the rationale for choosing this topic? Why is it important, especially considering that improvements in cardiovascular health could have more significant effects on life-threatening conditions such as coronary artery or cerebrovascular diseases?

7. Given the potential recall bias associated with self-reported data, did you consider validating the gallstone diagnoses with clinical records, or was this not feasible due to the limitations of the dataset?

8. Please provide p-values for each factor in Table 2 to better understand the statistical significance of the findings.

9. Did you check the data for normal distribution, and which tests were used for the univariate analyses presented in Table 1?

Reviewer #2: General comments:

- Why did the authors not use the latest NHANES version?? The authors could pool the different cycles of NHANES data and analyze them.

The rationale for conducting the study is valid and the study is well conducted.

All sections including introductions, method, results, and discussion are well-written with proper details.

The tables and pictures are well-defined.

**********

6. PLOS authors have the option to publish the peer review history of their article (what does this mean?). If published, this will include your full peer review and any attached files.

If you choose “no”, your identity will remain anonymous but your review may still be made public.

**Do you want your identity to be public for this peer review?** For information about this choice, including consent withdrawal, please see our Privacy Policy.

Reviewer #1: **Yes: **Mohammad Mehdi Mehrabi Nejad

Reviewer #2: No

**********

[NOTE: If reviewer comments were submitted as an attachment file, they will be attached to this email and accessible via the submission site. Please log into your account, locate the manuscript record, and check for the action link "View Attachments". If this link does not appear, there are no attachment files.]

While revising your submission, please upload your figure files to the Preflight Analysis and Conversion Engine (PACE) digital diagnostic tool, https://pacev2.apexcovantage.com/. PACE helps ensure that figures meet PLOS requirements. To use PACE, you must first register as a user. Registration is free. Then, login and navigate to the UPLOAD tab, where you will find detailed instructions on how to use the tool. If you encounter any issues or have any questions when using PACE, please email PLOS at figures@plos.org. Please note that Supporting Information files do not need this step.

# Author comment: Author response to Decision Letter 0

**DOI:** 10.1371/journal.pone.0312857.r002

18 Sep 2024

Dear Editor and Reviewers,

Thank you for your giving us an opportunity to revise our manuscript. On behalf of all the contributing authors, I would like to express our sincere appreciation of your letter and reviewers’ constructive comments concerning our manuscript entitled “Associations between Life’s essential 8 and gallstones among US adults: a cross-sectional study from NHANES 2017-2018” (PONE-D-24-20718). These opinions help to improve the academic rigor of our article. We have studied these comments carefully and made modifications which we hope meet with your approval.

Revised portions are marked red in the manuscript. All comments are laid out below in italicized font and specific concerns have been numbered. Point-by-point responses to all nice reviewers are listed below in blue.

All authors have read and approved the re-submission of the manuscript. If you have any questions, please let me know.

Thank you for your consideration of our paper and we are looking forward to hearing from you.

Sincerely yours,

Lanyu Chen

chenlanyu@163.com

--------------------------

Response to Reviewer 1

Reviewer 1:

The manuscript provides valuable insights into the relationship between cardiovascular health and gallstone prevalence. Addressing the following points could enhance the study's validity and make a significant contribution to the field:

Comment 1. The cross-sectional design of the study limits causal inference. The observed associations do not confirm that improvements in LE8 scores lead to reduced gallstone prevalence. Therefore, be cautious in the conclusions and choice of language used.

Response 1. Thank you very much for your professional advice. It was our oversight to overstate the relationship between LE8 scores and gallstones in certain sections of the manuscript. We have reviewed numerous NHANES-based articles and made the corrections (line 43-44 & 347-351). We sincerely hope this revised manuscript will meet your requirements. 

Comment 2. The reliance on self-reported data for diagnosing gallstones may introduce misclassification bias, as gallstones could be underreported or inaccurately diagnosed, impacting the study's internal validity.

Response 2. Thank you very much for your comment. Indeed, as you have noted, the reliance on self-reported data for diagnosing gallstones may introduce bias. However, since 1999, self-reporting has been the sole method for diagnosing gallstones in NHANES, and existing research on gallstones has also relied on this approach (Hepatology. 2023;77(6):1882-1895; Lipids Health Dis. 2024;23(1):203). Furthermore, although the prevalence may be underestimated, the missing data is random and is unlikely to have a substantial impact on the results we have described. We have addressed this limitation in the Discussion section and hope for your understanding.

Comment 3. The robustness of the findings could be improved by exploring additional potential confounders, such as parity status, which is a significant factor.

Response 3. Thank you very much for your insightful feedback. Your comments help us identify some shortcomings in adjusting for confounding factors. After reviewing studies related to LE8 and gallstones (Hepatology. 2023;77(6):1882-1895; J Transl Med. 2022;20(1):616), we incorporate additional confounding factors that need adjustment. Specifically, we adjust for the number of pregnancies in our statistical analysis for female participants. The updated values have been added to the Results section, our conclusions remain largely consistent with those previously reported, with some differences discussed in it (line 237-248). Detailed data we add in schedules and charts to upload (Table S2, Table S3, Table S4, Table S5 , Table S6 and Fig S1).

Comment 4. How do the demographic characteristics of the NHANES sample compare to the broader U.S. population? What are the implications for the generalizability of your findings?

Response 4. Thank you very much for your professional advice. NHANES is a national cross-sectional survey that collects information every two years from residents, non-institutionalized U.S. adults and children. Its complex sampling design ensures that the data are nationally representative. Our analysis has been conducted in strict adherence to established guidelines. While there may still be some differences compared to broader U.S. populations, our findings could provide valuable insights into the relationship between cardiovascular health and gallstone incidence. We have provided the necessary introduction in the Methods section and hope for your understanding.

Comment 5. How might the findings of this study influence clinical guidelines or public health policies concerning gallstone prevention?

Response 5. Thank you very much for your comment. Our results suggest that cardiovascular health may be a potential risk factor for the occurrence of gallstones. Identifying these driving factors could assist governments and health agencies in developing targeted health policies, optimizing the allocation of medical resources, and improving the efficiency and quality of healthcare services. Additionally, recognizing these risk factors can aid in predicting future health trends, thereby supporting policymakers and researchers in formulating long-term health plans and response strategies.

Comment 6. Could you elaborate on the rationale for choosing this topic? Why is it important, especially considering that improvements in cardiovascular health could have more significant effects on life-threatening conditions such as coronary artery or cerebrovascular diseases?

Response 6. Thank you very much for your comment. Please allow us to clarify the significance of our research. As you mentioned, cardiovascular health is indeed more prominently associated with life-threatening conditions such as coronary artery disease and cerebrovascular diseases, yet, research in these areas is already quite extensive. Gallstones are also commonly believed to be related to cardiovascular risk factors, as the formation of gallstones and atherosclerosis may share critical pathways, but this knowledge remains limited. We aim to provide our own insights into this relationship. Moreover, symptoms such as biliary colic, angina pectoris, and gallbladder-heart syndrome are critical to differentiate clinically. We hope to draw attention to the relationship between these conditions for clinicians. The LE8, as a novel metric for assessing cardiovascular health, is believed to have the potential to predict various diseases. Thus, we conducted this study with the hope of offering new perspectives on the relationship between cardiovascular health and gallstone incidence. We sincerely hope for your understanding.

Comment 7. Given the potential recall bias associated with self-reported data, did you consider validating the gallstone diagnoses with clinical records, or was this not feasible due to the limitations of the dataset?

Response 7. Thank you very much for your professional comment, and we fully agree with your viewpoints. We acknowledge that relying on self-reported data to diagnose gallstones may introduce bias. However, as mentioned earlier, the limitations of the database have constrained the use of more precise methods. The specific limitations have been detailed in the Discussion section. Our team is currently working on collecting more accurate data, and we hope to address these limitations in the future. 

Comment 8. Please provide p-values for each factor in Table 2 to better understand the statistical significance of the findings.

Response 8. We sincerely apologize for the lack of rigor. All P-values have been added in the revised manuscript.

Comment 9. Did you check the data for normal distribution, and which tests were used for the univariate analyses presented in Table 1?

Response 9. Thank you very much for your professional comment. First, we apologize for not specifying the testing methods used. We have now provided additional details in the Methods section (line 133-135). Secondly, we conducted a normality test and found that LE8 does not follow a normal distribution. However, we continue to present it using means and standard deviations for the following reasons:

1) Due to the unique characteristics of complex sampling, the methods and significance of normality testing are still debated. Most studies use means and standard deviations directly when the sample size is sufficiently large.

2) For large samples with complex sampling, even minor variations can affect data normality. To address this, we performed a distribution curve fitting, and the curve closely approximates a normal distribution.

3) LE8 is an index derived from eight parameters through complex calculations, which is not suitable for normality transformation.

Consequently, we have chosen to represent the continuous data using means and confidence intervals (CIs). We sincerely hope for your understanding.

-------------------------------

Response to Reviewer 2

Comment 1. Why did the authors not use the latest NHANES version?? The authors could pool the different cycles of NHANES data and analyze them.

Response 1. Thank you for your positive feedback, and we are delighted to receive your recognition. Diagnosis of gallstones was only performed after 2017. However, given the potential impact of the COVID-19 pandemic on cardiovascular health, we have chosen to use only data from 2017-2018. We sincerely hope for your understanding.

Attachment Submitted filename: Response to Reviewers.docx

# Peer review: Decision Letter 1

**DOI:** 10.1371/journal.pone.0312857.r003

**Authors:** Mohammad Reza Fattahi

15 Oct 2024

Associations between Life’s essential 8 and gallstones among US adults: A cross-sectional study from NHANES 2017-2018

PONE-D-24-20718R1

Dear Dr. Chen

We’re pleased to inform you that your manuscript has been judged scientifically suitable for publication and will be formally accepted for publication once it meets all outstanding technical requirements.

Within one week, you’ll receive an e-mail detailing the required amendments. When these have been addressed, you’ll receive a formal acceptance letter and your manuscript will be scheduled for publication.

An invoice will be generated when your article is formally accepted. Please note, if your institution has a publishing partnership with PLOS and your article meets the relevant criteria, all or part of your publication costs will be covered. Please make sure your user information is up-to-date by logging into Editorial Manager at Editorial Manager® and clicking the ‘Update My Information' link at the top of the page. If you have any questions relating to publication charges, please contact our Author Billing department directly at authorbilling@plos.org.

If your institution or institutions have a press office, please notify them about your upcoming paper to help maximize its impact. If they’ll be preparing press materials, please inform our press team as soon as possible -- no later than 48 hours after receiving the formal acceptance. Your manuscript will remain under strict press embargo until 2 pm Eastern Time on the date of publication. For more information, please contact onepress@plos.org.

Kind regards,

Mohammad Reza Fattahi, M.D., M.P.H.

Academic Editor

PLOS ONE

Additional Editor Comments (optional):

Reviewers' comments:

Reviewer's Responses to Questions

**Comments to the Author**

1. If the authors have adequately addressed your comments raised in a previous round of review and you feel that this manuscript is now acceptable for publication, you may indicate that here to bypass the “Comments to the Author” section, enter your conflict of interest statement in the “Confidential to Editor” section, and submit your "Accept" recommendation.

Reviewer #1: All comments have been addressed

Reviewer #3: All comments have been addressed

**********

2. Is the manuscript technically sound, and do the data support the conclusions?

The manuscript must describe a technically sound piece of scientific research with data that supports the conclusions. Experiments must have been conducted rigorously, with appropriate controls, replication, and sample sizes. The conclusions must be drawn appropriately based on the data presented. 

Reviewer #1: Yes

Reviewer #3: Yes

**********

3. Has the statistical analysis been performed appropriately and rigorously? 

Reviewer #1: Yes

Reviewer #3: Yes

**********

4. Have the authors made all data underlying the findings in their manuscript fully available?

The PLOS Data policy requires authors to make all data underlying the findings described in their manuscript fully available without restriction, with rare exception (please refer to the Data Availability Statement in the manuscript PDF file). The data should be provided as part of the manuscript or its supporting information, or deposited to a public repository. For example, in addition to summary statistics, the data points behind means, medians and variance measures should be available. If there are restrictions on publicly sharing data—e.g. participant privacy or use of data from a third party—those must be specified.

Reviewer #1: Yes

Reviewer #3: Yes

**********

5. Is the manuscript presented in an intelligible fashion and written in standard English?

PLOS ONE does not copyedit accepted manuscripts, so the language in submitted articles must be clear, correct, and unambiguous. Any typographical or grammatical errors should be corrected at revision, so please note any specific errors here.

Reviewer #1: Yes

Reviewer #3: Yes

**********

6. Review Comments to the Author

Please use the space provided to explain your answers to the questions above. You may also include additional comments for the author, including concerns about dual publication, research ethics, or publication ethics. (Please upload your review as an attachment if it exceeds 20,000 characters)

Reviewer #1: Thank you for revising the manuscript. All comments are addressed appropriately and it is suitable for publication now.

Reviewer #3: All comments have been addressed properly, and can be accepted for publication, I have no more comments on this manuscript.

**********

7. PLOS authors have the option to publish the peer review history of their article (what does this mean?). If published, this will include your full peer review and any attached files.

If you choose “no”, your identity will remain anonymous but your review may still be made public.

**Do you want your identity to be public for this peer review?** For information about this choice, including consent withdrawal, please see our Privacy Policy.

Reviewer #1: No

Reviewer #3: No

**********

# Editor report: Acceptance letter

**DOI:** 10.1371/journal.pone.0312857.r004

**Authors:** Mohammad Reza Fattahi

21 Oct 2024

PONE-D-24-20718R1 

PLOS ONE

Dear Dr. chen, 

I'm pleased to inform you that your manuscript has been deemed suitable for publication in PLOS ONE. Congratulations! Your manuscript is now being handed over to our production team.

At this stage, our production department will prepare your paper for publication. This includes ensuring the following:

* All references, tables, and figures are properly cited

* All relevant supporting information is included in the manuscript submission,

* There are no issues that prevent the paper from being properly typeset

If revisions are needed, the production department will contact you directly to resolve them. If no revisions are needed, you will receive an email when the publication date has been set. At this time, we do not offer pre-publication proofs to authors during production of the accepted work. Please keep in mind that we are working through a large volume of accepted articles, so please give us a few weeks to review your paper and let you know the next and final steps. 

Lastly, if your institution or institutions have a press office, please let them know about your upcoming paper now to help maximize its impact. If they'll be preparing press materials, please inform our press team within the next 48 hours. Your manuscript will remain under strict press embargo until 2 pm Eastern Time on the date of publication. For more information, please contact onepress@plos.org.

If we can help with anything else, please email us at customercare@plos.org.

Thank you for submitting your work to PLOS ONE and supporting open access. 

Kind regards, 

PLOS ONE Editorial Office Staff

on behalf of

Dr. Mohammad Reza Fattahi 

Academic Editor

PLOS ONE
